# HLA-DR Expression in Natural Killer Cells Marks Distinct Functional States, Depending on Cell Differentiation Stage

**DOI:** 10.3390/ijms25094609

**Published:** 2024-04-23

**Authors:** Sofya A. Kust, Maria O. Ustiuzhanina, Maria A. Streltsova, Pavel V. Shelyakin, Maxim A. Kryukov, Gennady V. Lutsenko, Anna V. Sudarikova, Ekaterina M. Merzlyak, Olga V. Britanova, Alexandr M. Sapozhnikov, Elena I. Kovalenko

**Affiliations:** 1 Shemyakin & Ovchinnikov Institute of Bioorganic Chemistry, Russian Academy of Sciences, 117997 Moscow, Russia; sonya.erokhina@gmail.com (S.A.K.); mashaust1397@gmail.com (M.O.U.); mstreltsova@mail.ru (M.A.S.); mkriukov.job@gmail.com (M.A.K.); gvlut@mail.ru (G.V.L.); ekaterin99@mail.ru (E.M.M.); olbritan@gmail.com (O.V.B.); amsap@mail.ru (A.M.S.); 2 Abu Dhabi Stem Cells Center, Abu Dhabi 4600, United Arab Emirates; pavel.s@adscc.ae; 3 Federal State Autonomous Institution, N.N. Burdenko National Medical Research Center of Neurosurgery, the Ministry of Health of the Russian Federation, 125047 Moscow, Russia; negreeva.ann@gmail.com; 4 Institute of Translational Medicine, Pirogov Russian National Research Medical University, 117997 Moscow, Russia

**Keywords:** NK cells, HLA-DR, RNAseq, CIITA, metabolism, IL-21, IFNg, STAT1, STAT3, ERK1/2

## Abstract

HLA-DR-positive NK cells, found in both healthy individuals and patients with different inflammatory diseases, are characterized as activated cells. However, data on their capacity for IFNγ production or cytotoxic response vary between studies. Thus, more precise investigation is needed of the mechanisms related to the induction of HLA-DR expression in NK cells, their associations with NK cell differentiation stage, and functional or metabolic state. In this work, HLA-DR-expressing NK cell subsets were investigated using transcriptomic analysis, metabolic activity assays, and analysis of intercellular signaling cascades. We demonstrated that HLA-DR^+^CD56^bright^ NK cells were characterized by a proliferative phenotype, while HLA-DR^+^CD56^dim^ NK cells exhibited features of adaptive cells and loss of inhibitory receptors with increased expression of MHC class II trans-activator CIITA. The activated state of HLA-DR-expressing NK cells was confirmed by higher levels of ATP and mitochondrial mass observed in this subset compared to HLA-DR^−^ cells, both ex vivo and after stimulation in culture. We showed that HLA-DR expression in NK cells in vitro can be induced both through stimulation by exogenous IL-2 and IL-21, as well as through auto-stimulation by NK-cell-produced IFNγ. At the intracellular level, HLA-DR expression depended on the activation of STAT3- and ERK1/2-mediated pathways, with subsequent activation of isoform 3 of the transcription factor CIITA. The obtained results broaden the knowledge about HLA-DR-positive NK cell appearance, diversity, and functions, which might be useful in terms of understanding the role of this subset in innate immunity and assessing their possible implications in NK cell-based therapy.

## 1. Introduction

HLA-DR-positive NK cells can be found in healthy donors both in the blood and in various immunocompetent organs: spleen, liver, lymph nodes, and adenoids. More and more data support the fact that HLA-DR-expressing NK cell percentage increases in the blood and tissues under various pathological conditions, such as HIV infection [1], various autoimmune diseases [2], tuberculosis [3], and COVID-19 [4,5]. At the same time, the upregulation of HLA-DR expression has been shown for NK cells expanded in the presence of various stimuli or in mixed lymphocyte reactions in vitro [6,7]. A well-known function of the HLA-DR molecule, a type of MHC class II, is participation in antigen presentation, and, therefore, it is highly expressed in professional antigen-presenting cells [8]. It has also been shown that HLA-DR^+^ NK cells are able to act as antigen-presenting cells: activate in IFNγ and TNFα production and the degranulation of CD4^+^ T cells [7,9,10,11]. Moreover, ex vivo and stimulated HLA-DR-expressing NK cells commonly demonstrate higher cytokine production and cytotoxic activity compared to HLA-DR-negative NK cells, making HLA-DR an activation marker [11,12,13]. At the same time, little is known about the mechanism of induction of expression of this molecule in NK cells, although such data have been published for T cells and B cells [8].

It has been shown in our previous work, as well as by other authors, that HLA-DR expression on NK cells can be significantly increased in vitro upon stimulation with IL-2 and IL-21, especially with membrane-bound IL-21 on the surface of K562 feeder cells [7,12,14]. According to one of the studies, this process is regulated through the STAT3 pathway [7]. At the same time, activated HLA-DR-positive NK cells are often characterized by the increased production of IFNγ [7]. Using clonal cultures, we showed a direct correlation between the amount of IFNγ produced and the intensity of HLA-DR expression [15]. The relationship between IFNγ stimulation and an increase in the expression of MHC class II, mainly through the activation of the JAK-STAT1 pathway, has been shown a while ago for a number of human cell types [16]; however, no similar data have been presented for NK cells. Considering the increase in IFNγ production by NK cells under the influence of IL-21 and other cytokines [17], it can be assumed that the induction of HLA-DR expression in NK cells can occur both via exogenous stimulation and autostimulation with IFNγ, involving the transcription factor STAT1 or other second messengers. Receptors for IFNγ are present in almost all cells of the body, including NK cells [18].

At the transcriptional level, the regulation of HLA-DR expression, as well as all MHC II molecules, is carried out by a specialized complex of cis-regulatory elements that form the enhanceosome and a class II transactivator, CIITA, that does not directly contact DNA but is, in fact, the main regulator of MHC II expression. Enhanceosome components are expressed at approximately the same level in all cells of the body, while CIITA expression depends on the cell type, the presence of stimulating/inhibitory cytokines in the environment, and the stage of cell differentiation and clearly correlates with the level of MHC II expression [19]. CIITA can be expressed from one of three promoters, the activity of which depends on the cell type [20,21]. In cells constitutively expressing MHC II, and therefore CIITA, promoters pI (macrophages and induced dendritic cells) and pIII (B cells and plasmacytoid dendritic cells) operate in relation to the expression of CIITA isoforms 1 and 3, respectively. In activated T cells, the induction of MHC II expression also occurs through the pIII promoter [19]. In a variety of other cell types, the upregulation of MHC II expression requires the activation of the inducible promoter pIV, i.e., by IFNγ via a STAT1-dependent pathway [16]. There is scarce information about other cytokines capable of stimulating CIITA expression. However, it is known that CIITA is one of the targets of ERK1/2, a transcription factor and terminal kinase of the MAPK kinase pathway, and is common for many activation signals [22]. The mechanism of CIITA activation and, thus, the induction of MHC II expression in NK cells has not been described in the literature yet.

The functional activity of NK cells, including the production of IFNγ, is inextricably linked with metabolic changes [23]. In resting NK cells, metabolic activity is low, with little involvement of glycolysis and oxidative phosphorylation. However, in activated NK cells, the intensity of these processes upregulates significantly, glucose consumption increases, primarily for glycolysis, and mitochondrial mass and ATP production increase too [24]. At the same time, the relationship between HLA-DR expression on NK cells and their metabolic characteristics is currently unknown.

Presently, the molecular basis underlying HLA-DR^+^ NK cell biology needs to be investigated. In this work, we determined the impact of molecular pathways, including Jak1/3-STAT1, Ras/Raf/Mek/ERK1/2, JAK1/3-STAT3, and signaling from IL-21R and IFNγR on HLA-DR expression in NK cells. In addition, we have investigated transcriptomic and metabolic differences between HLA-DR-positive and negative NK cells.

## 2. Results

### 2.1. HLA-DR Expression in NK Cells Is Regulated by Isoform 3 of the Transcription Factor CIITA

**Figure 1:**
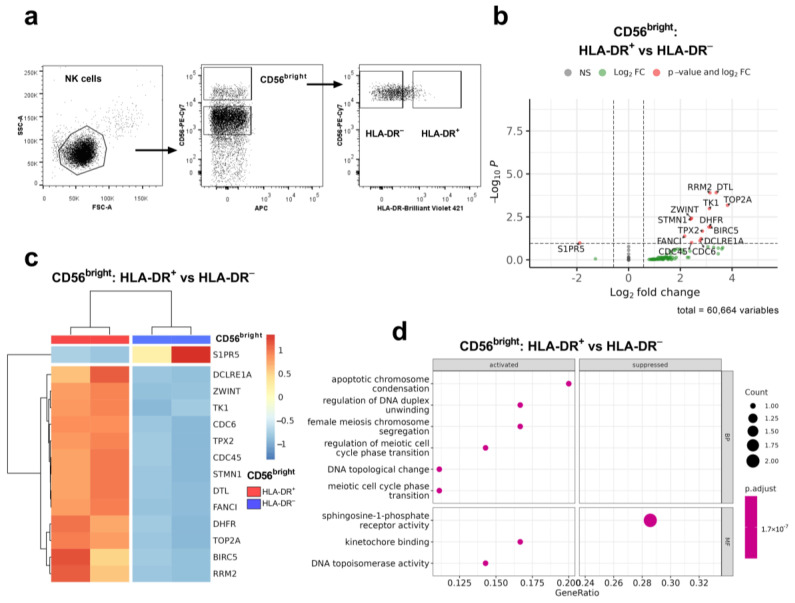
The figure shows the transcriptional profiling of HLA-DR^+^ and HLA-DR^−^ CD56^bright^ NK cells from donor 1. (**a**) Scheme of gating of CD56^bright^ NK cells for sorting and following sequencing; (**b**) Volcano plot comparing HLA-DR^+^ with HLA-DR^−^ CD56^bright^ NK cells significant upregulated and downregulated genes are labeled. (**c**) Unsupervised hierarchical clustering of differentially expressed genes (LFC > 0.58, *p* adj < 0.1): each column represents the samples of CD56^bright^ NK cells (HLA-DR^+^ (red), HLA-DR^−^ (blue)) from two donors and each row represents a gene. The heatmap indicates the level of gene expression: red is increased, and blue indicates decreased expression; (**d**) Dotplot of GeneOntology gene set enrichment analysis of HLA-DR^+^ vs. HLA-DR^−^ CD56^bright^ NK cells, where the *y*-axis represents GO molecular pathways (Biological Processes (BP) Molecular Functions (MF)), separated by sign and *x*-axis gene ratio. The greater the size of a circle, the greater the number of genes involved in a pathway. The circles are colored based on *p*-adjusted values.

To evaluate the differences between HLA-DR^+^ and HLA-DR^−^ NK cells at the transcriptomic level, we conducted a transcriptome analysis of sorted NK cell fractions ex vivo. NK cells were isolated from two healthy donors; each subset was sorted into two replicates and subjected to RNA sequencing. We started with the analysis of less mature HLA-DR^+^ and HLA-DR^−^ CD56^bright^ NK cells from donor 1 (Figure 1a).

Via unbiased analyses of differentially expressed genes (DEGs) and gene ontology (GO) network pathways, we identified that the HLA-DR^+^CD56^bright^ subset is characterized by the enrichment of processes associated with cell proliferation and apoptosis resistance, in contrast to the HLA-DR^−^CD56^bright^ subset (Figure 1b–d). Genes upregulated in HLA-DR^+^CD56^bright^ NK cells compared to HLA-DR^−^CD56^bright^ NK cells are involved in replication (*TOP2A*, *TK1*, *CDC6*, *RRM2*, *DHFR*, *CDC45*, and *DTL*), the regulation of the transition between cell cycle stages S-G2-M (*DCLRE1A*), the regulation of the mitosis process (*STMN1*, *ZWINT*), and the inhibition of apoptosis (*BIRC5*). These results suggest that HLA-DR expression in CD56^bright^ NK cells is associated primarily with their proliferative activity and thus is upregulated in actively proliferating cells.

**Figure 2:**
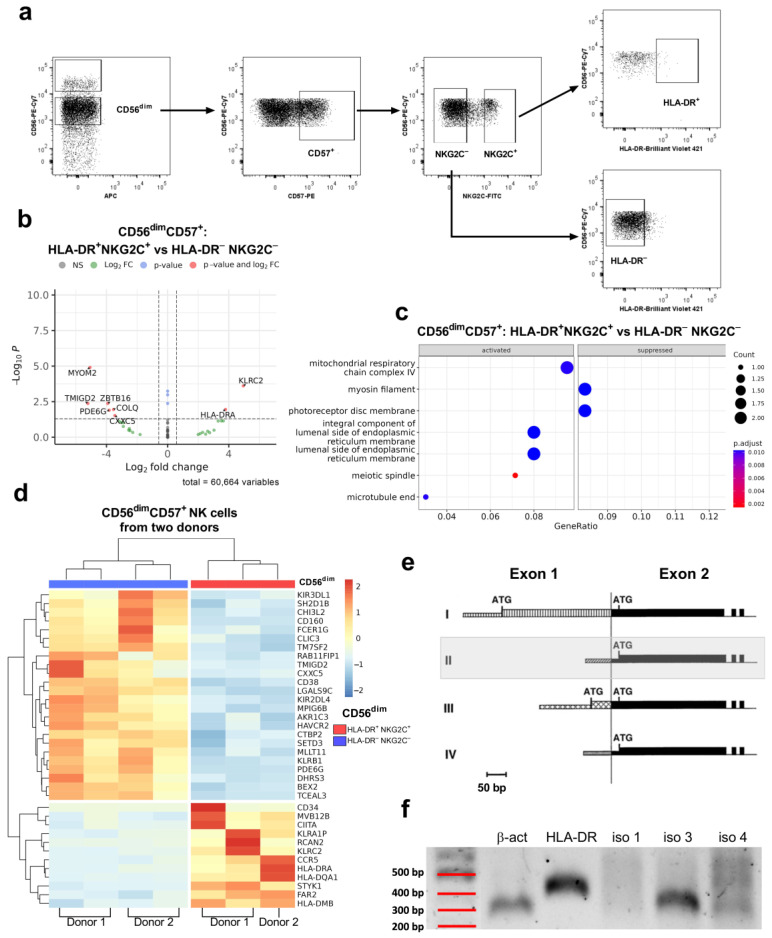
The figure shows the transcriptional profiling of HLA-DR^+^NKG2C^+^ with HLA-DR^−^NKG2C^−^ CD56^dim^CD57^+^ NK cells. (**a**) Scheme of gating of CD56^dim^CD57^+^ NK cells for sorting and following sequencing in example of donor 1; (**b**) volcano plot comparing HLA-DR^+^NKG2C^+^ with HLA-DR^−^NKG2C^−^ CD56^dim^CD57^+^ NK cells of donor 1; significant upregulated and downregulated genes are labeled; (**c**) Dotplot of GeneOntology gene set enrichment analysis of HLA-DR^+^NKG2C^+^ vs. HLA-DR^-^NKG2C^−^ CD57^+^CD56^dim^ NK cells of donor 1, where *y*-axis represents GO molecular pathway cellular component, separated by sign and *x*-axis gene ratio. The greater the size of a circle, the greater the number of genes involved in a pathway. The circles are colored based on *p*-adjusted values. (**d**) Unsupervised hierarchical clustering of a part of differentially expressed genes (LFC > 0.58, *p* adj < 0.05): each column represents the samples of CD56^dim^CD57^+^ NK cells (HLA-DR^+^NKG2C^+^ (red), HLA-DR^−^NKG2C^−^ (blue)) from two donors and each row represents a gene. The heatmap indicates the level of gene expression: red is increased, and blue indicates decreased expression; (**e**) Schematic illustration of the structure of mRNA (the first two exons) of the CIITA isoforms (modified) [19]. Narrow and wide stripes indicate untranslated and translated areas, respectively; ATG—possible translation initiation sites; isoform II isolated gray as not participating in this study; (**f**) result of RT-PCR analysis, demonstrating expression of CIITA protein isoform 3 in NK cells; β-act—β-actin (positive control); and iso—isoform.

Previously, we showed that despite the fact that HLA-DR^+^ cells often predominate in the CD56^bright^ NK cell subpopulation in healthy people, in a group of donors, they were enriched in the terminally differentiated CD56^dim^CD57^+^ subset [12]. Increased HLA-DR expression was also registered in hCMV-associated adaptive NK cells CD57^+^NKG2C^+^ [10,12,25,26]. Therefore, highly differentiated HLA-DR^+^NKG2C^+^ and HLA-DR^−^NKG2C^−^ CD57^+^CD56^dim^ NK cells were compared (Figure 2a).

For HLA-DR^+^NKG2C^+^CD57^+^CD56^dim^ NK cells of the same donor 1, the upregulated genes corresponded to the phenotype according to which these cells were sorted (Figure 2a). Downregulated genes include the genes encoding transcriptional factor PLZF (*ZBTB16*), activating receptor (*TMIGD2*), and intracellular proteins (*MYOM2*, *COLQ*, *CXXC5*, and *PDE6G*) (Figure 2b). Some of these genes were noticed to characterize less differentiated canonical NK cells [27,28]. GeneOntology gene set enrichment analysis revealed the activation of mitochondrial respiratory chain complex IV in HLA-DR^+^NKG2C^+^ cells compared to HLA-DR^−^NKG2C^−^ cells in the CD57^+^CD56^dim^ subset (Figure 2c).

The analysis of donor 2 revealed that one of the replicas of the CD56^dim^CD57^+^HLA-DR^+^NKG2C^+^ subset was too different from other samples, and, thus, was excluded from the analysis (Supplementary Figure S1a). The remaining samples of CD56^dim^ NK cells from both donors were combined for joint DEG analysis (Supplementary Figure S1b,c). For CD56^dim^ NK cells, the downregulated genes in the HLA-DR^+^ subset included activating and inhibitory receptors (CD160, KLRB1, KIR2DL4, and KIR3DL1), signal molecules (*SH2D1B* (EAT-2), *FCER1G* (FceR1g)), glycoprotein CD38, and some other genes (Figure 2d). The upregulated genes included genes encoding NKG2C (*KLRC2*), antigen-presenting HLA class II molecules (*HLA-DRA*, *HLA-DQA1*, and *HLA-DMB*), regulatory factor CIITA, chemokine receptor CCR5, marker of NK progenitor cells CD34 and some more genes, encoding intracellular proteins: *STYK1*, *FAR2*, *MVB12B*, and *RCAN2* (Figure 2d). The overall transcriptional profile of HLA-DR^+^NKG2C^+^CD57^+^CD56^dim^ NK cells confirmed that these cells represent an adaptive cell subset. Moreover, HLA-DR characterizes less mature/educated NK cells.

As mentioned in the introduction, CIITA, which was identified among differentially upregulated genes in the HLA-DR^+^NKG2C^+^CD57^+^CD56^dim^ subset, is the main regulatory factor for MHC class II expression. The four isoforms of CIITA, expressed in distinct cell types, differ in the structure of the first exon and the untranslated region upstream of it (Figure 2e) [19]. To determine which isoform is present in NK cells and is involved in the induction of HLA-DR expression, primers were selected for the contact site of exons 1 and 2 of isoforms I, III, and IV for RT-PCR analysis. NK cells were pre-cultivated with IL-2^+^K562-mbIL21 for 6 days to induce HLA-DR expression, then subjected to PCR. The resulting amplicons were visualized using gel electrophoresis. Figure 2f shows the PCR results of a representative experiment, indicating that activated NK cells express isoform III of the CIITA regulator. Thus, NK cells express the same isoform as T cells upon activation, which is likely a consequence of their origin from common progenitor cells.

### 2.2. HLA-DR Expression in NK Cells Is Associated Not Only with IFNg Production, but Also with Metabolic Activation

The activation of NK cells and, in particular, the increased production of IFNγ is associated with metabolic changes: upregulated glycolysis and OXPHOS, increased nutrient uptake [24,29,30]. Since the acquisition of HLA-DR expression is clearly associated with NK cell activation and is closely related to IFNγ production, we decided to evaluate the metabolic status of HLA-DR-expressing NK cells. Transcriptome analysis described in the previous section showed the enriched genes involved in the activation of mitochondrial respiratory chain complex IV in HLA-DR^+^NKG2C^+^ cells compared to HLA-DR^−^NKG2C^−^ cells in CD57^+^CD56^dim^ subset (Figure 2c). Moreover, as long as the activity of metabolic processes can also be regulated on various post-transcriptional levels, we have additionally conducted an analysis of the ATP content and the volume of mitochondrial mass in HLA-DR^+^ и HLA-DR^–^ NK cell subsets, freshly isolated or pre-stimulated in vitro.

**Figure 3:**
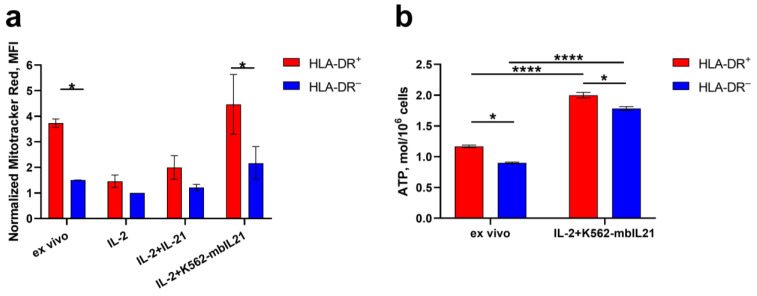
The figure shows a comparison of HLA-DR^+^ metabolic activity and HLA-DR^−^ NK cells. (**a**) Normalized mitochondrial mass volume of HLA-DR^+^ and HLA-DR^–^ NK cells measured using Mitotracker Red fluorescence intensity immediately after isolation and after 6 days of cultivation with IL-2, or IL-2 and IL-21, or IL-2 and K562-mbIL21 feeder cells (*n* = 3). (**b**) ATP content in HLA-DR^+^ and HLA-DR^−^ subpopulations immediately after isolation and after 6 days of cultivation with IL-2 and K562-mbIL21 (*n* = 6). Results are presented as mean ± SD. The statistical significance of the results was assessed using a two-way ANOVA with Sidak’s multiple correction. * *p* < 0.05, **** *p* < 0.0001.

According to ex vivo data, the volume of mitochondrial mass in HLA-DR-positive NK cells was higher than in HLA-DR-negative ones (Figure 3a), indicating intense metabolic activity of circulating HLA-DR^+^ NK cells. When stimulated with IL-2 or a combination of IL-2 and soluble IL-21, mitochondrial mass levels decreased in both subpopulations and leveled off between them (Figure 3a). Most likely, this is explained by NK cell proliferation stimulated by IL-2 and IL-21: during cell division, the number of mitochondria in each daughter cell decreases, and in HLA-DR-positive cells, their number decreases more strongly since they proliferate more intensely [12]. When NK cells were stimulated with IL-2 and K562-mbIL21 feeder cells, mitochondrial mass levels remained unchanged compared to ex vivo samples and were also increased in the HLA-DR^+^ subset compared to HLA-DR^−^ (Figure 3a). Apparently, this activation method not only triggers intensive expansion of NK cells but also promotes more intense biosynthesis, which allows cells to maintain the number of mitochondria at the same level even after active proliferation.

To compare the ATP production intensity in HLA-DR-positive and negative NK cells, which were freshly isolated and incubated for 6 days in the presence of IL-2 and IL-21, NK cells were sorted into HLA-DR^+^ and HLA-DR^−^ subpopulations and then treated with a lysis agent and subjected to ATP content measurements via bioluminescence. ATP production in HLA-DR-positive NK cells was higher than in HLA-DR-negative ones, both immediately after isolation and after 6 days of culture with IL-2+IL-21 (Figure 3b). In addition, ATP production in both subsets increased on day 6 of activation compared to ex vivo levels (Figure 3b).

### 2.3. HLA-DR Expression in NK-Cells Depends Both on Exogenous IL-21 and Endogenous IFNγ

To elucidate the mechanism underlying the induction of HLA-DR expression in NK cells and check the possible role of endogenously produced IFNγ in this process, we performed an experiment with cytokine-receptor blockade. During in vitro stimulation of NK cells with IL-2+Il-21, an IL-21 inhibitor (a soluble form of the IL-21 receptor, rhIL-21R) or a blocking antibody to the type 1 interferon receptor (anti-IFNγR1) was added to the medium. Both IL-2 and IL-21 are shown to induce IFNγ production via NK cells, although their combination is considered the most effective [7,17,31].

**Figure 4:**
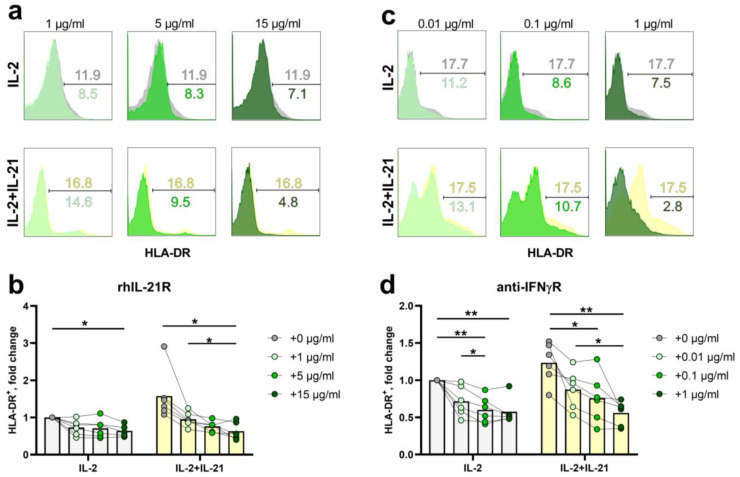
The figure shows the effect of rhIL-21R and anti-IFNγR1 blockers on HLA-DR expression on NK cells upon stimulation with IL-2 ± IL-21. (**a**,**c**) Representative cytometric data and (**b**,**d**) summarized data for 6 experiments, demonstrating the effect of rhIL-21R and antibodies to IFNγR1, accordingly, at the indicated concentrations on the percentage of HLA-DR positive NK cells in culture. Data are presented as mean with individual values with connectors normalized to values in IL-2-only controls. Statistical significance of the differences was assessed by repeated measures one-way ANOVA with Tukey’s multiple correction. * *p* < 0.05, ** *p* < 0.01.

When NK cells were stimulated with IL-2 and IL-21 for 6 days in the presence of rhIL-21R, HLA-DR expression gradually decreased with the increase in rhIL-21R dose, reaching significance at a concentration of 15 μg/mL (Figure 4a,b). In the samples stimulated with only IL-2, there was also a slight target-independent decrease in the proportion of HLA-DR^+^ cells with the addition of rhIL-21R, possibly caused by prolonged in vitro cultivation of cells (Figure 4a,b).

In the presence of antibodies to IFNγR1 at a concentration of 0.1 μg/mL and higher, a significant dose-dependent decrease in the proportion of HLA-DR-expressing NK cells was registered in samples stimulated with IL-2 and IL-21, compared to samples without a blocker or samples with lower blocker concentration (Figure 4c,d). A significant decrease in the percentage of HLA-DR^+^ NK cells was also observed in samples stimulated with IL-2 only, at all concentrations of the antibody (Figure 4c,d).

The obtained results indicate that, at least in part, the induction of HLA-DR expression in NK cells activated by cytokine stimulation occurs by triggering the synthesis of IFNγ, which, in turn, binds to IFNγ receptors on the surface of NK cells. This leads to auto-stimulation and the augmentation of HLA-DR expression via a positive feedback loop. Moreover, we demonstrate that IFNγ auto-stimulation occurs both upon exposure to IL-2 and IL-21.

### 2.4. STAT3- and ERK1/2-Mediated Signal Transduction Pathways Participate in HLA-DR Induction in NK Cells

It is known that the main pathway of signal transduction from IL-21R to the cell nucleus is the JAK1/3-STAT3 signaling pathway, while JAK1/3-STAT1 and Ras/Raf/Mek/ERK1/2 cascades are only mildly activated [14]. The signal from IFNγR is transmitted mainly through the JAK1/3-STAT1 pathway and also through the Ras/Raf/Mek/ERK1/2 kinase cascade [18,32].

To assess the participation of signaling pathways described above in the induction of HLA-DR expression, we performed a series of NK cell activation experiments in the presence of IL-2+IL21 and selective inhibitors of STAT1, STAT3 and ERK1/2—fludarabine phosphate, cryptotanshinone and FR180204, respectively. The concentrations and time intervals for adding the inhibitors were selected empirically based on recommendations in the literature and preliminary experiments [33,34]. The following inhibitor concentrations were used: 0.1 μg/mL, 1 μg/mL, 10 μg/mL for fludarabine phosphate and cryptotanshinone, 0.5 μg/mL, 5 μg/mL, 50 μg/mL for FR180204. Inhibitors were added on the third day of NK cell cultivation with the indicated stimuli, as HLA-DR expression begins to increase at this time point, according to our previous work [12].

**Figure 5:**
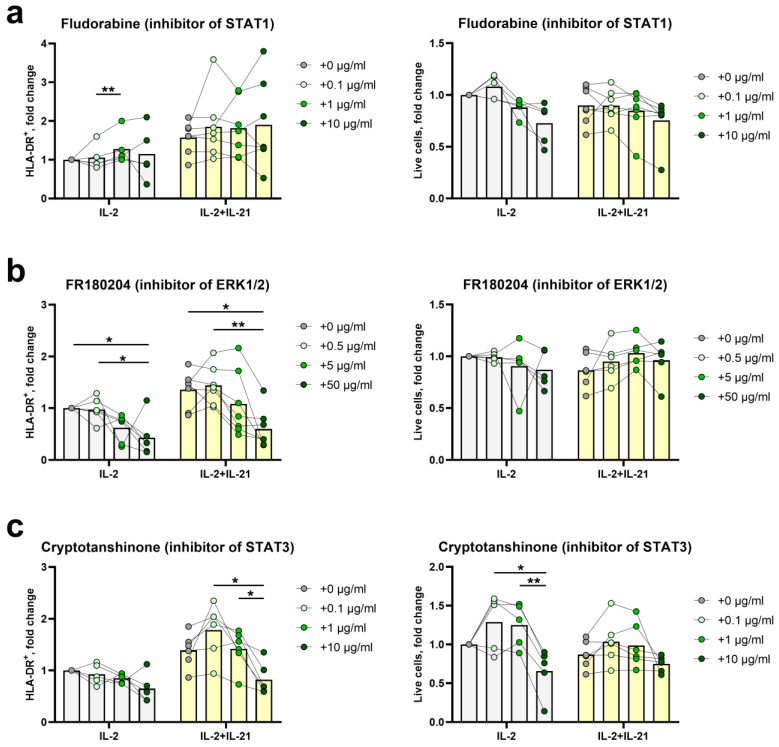
The figure shows the analysis of the participation of intracellular signaling molecules in the induction of HLA-DR expression in NK cells upon stimulation with IL-2 ± IL-21. (**a**) Effect of STAT1 inhibitor at the indicated concentrations on the level of HLA-DR expression on NK cells and viability of NK cells on the 5th day of stimulation. (**b**) Effect of ERK1/2 inhibitor at the indicated concentrations on the level of HLA-DR expression on NK cells and viability of NK cells on the 5th day of stimulation. (**c**) Effect of STAT3 inhibitor at the indicated concentrations on level of HLA-DR expression on NK cells and viability of NK cells on the 5th day of stimulation. (**a**–**c**) All samples without inhibitors (concentration 0 μg/mL) were supplemented with the corresponding amount of solvent DMSO. Summarized data from five experiments for IL-2 and seven for IL-2+IL-21 is presented as mean with individual values normalized to values in IL-2+DMSO controls. Samples from the same donor are linked. Statistical significance of the observed differences was assessed by repeated measures one-way ANOVA with Tukey’s multiple correction. * *p* < 0.05, ** *p* < 0.01.

As shown in Figure 5a, STAT1 inhibitor fludarabine did not cause a significant inhibition of HLA-DR expression in NK cells stimulated with IL-2 compared to the control sample IL-2+DMSO. On the contrary, a tendency to a slight increase in the expression was visible: when fludarabine was added at a concentration of 1 μg/mL, the proportion of HLA-DR^+^ NK cells was higher compared to the 0.1 μg/mL concentration. In samples additionally stimulated with IL-21, no significant differences were detected in the proportion of HLA-DR^+^ NK cells between samples with and without the inhibitor. Thus, it seems that the activation of the JAK1-STAT1 signaling pathway upon IFNγ-IFNγR ligation does not affect the expression of HLA-DR in NK cells, or, alternatively, compensatory pathways are actively involved, for example, Ras/Raf/Mek/ERK1/2.

In experiments with the ERK1/2 inhibitor, there was a tendency to a decrease in HLA-DR expression at 5 μg/mL concentration, and there was an almost two times decrease at 50 μg/mL concentration compared to the controls with DMSO, both in IL-2 and IL-2+IL-21-stimulated samples (Figure 5b). This indicates that the activation of the Ras/Raf/Mek/ERK1/2 signaling pathway, which is common between IFNγR, IL-21R and IL2R, is required for the induction of HLA-DR expression.

The addition of cryptotanshinone, the inhibitor of the transcription factor STAT3, upon stimulation with IL-2+IL-21 significantly decreased the expression of HLA-DR on the surface of NK cells at a concentration of 10 μg/mL, compared to samples with lower concentrations of the inhibitor. These data confirm the involvement of the JAK1/3-STAT3 signaling pathway, which is central for IL-21R signaling, in the induction of HLA-DR expression (Figure 5c). The observed effect is consistent with the previous findings on the role of the STAT3 factor in signal transduction from IL-21, with a subsequent increase in the expression of HLA-DR and CD86 in NK cells [7]. In samples with IL-2 only, cryptotanshinone had no effect on the proportion of HLA-DR^+^ cells (Figure 5c).

Thus, at the intracellular level, the induction of HLA-DR expression in NK cells upon stimulation with IL-2+IL-21 is mediated by the transcription factors ERK1/2 and STAT3, which apparently trigger the expression or regulate the activity of the main transcription regulator of MHC II-CIITA.

In addition to HLA-DR expression, the number of live NK cells was also assessed in all samples (Figure 5a–c). In all samples stimulated using IL-2^+^IL-21, NK cell survival was not significantly affected by the inhibitor at any concentration (Figure 5a–c). However, in IL-2-only samples, an increased death rate was registered at 10 μg/mL of cryptonshinone (Figure 5c). Possibly, high concentrations of the STAT3 inhibitor induce NK cell apoptosis, which cannot be overcome by a pro-survival signal from IL-2 alone. Overall, the survival data support the fact that the results on the influence of STAT1, STAT3 and ERK1/2 factors on the percentage of HLA-DR-positive NK cells were not obtained due to the selective death of certain cell subsets under the influence of inhibitors.

## 3. Discussion

The activation of NK cells is followed by the acquisition of HLA-DR expression. Despite a relatively low number of HLA-DR^+^ NK cells in human peripheral blood (6% on average), it is possible to expand the HLA-DR-expressing subset in vitro significantly (up to 80% of all NK cells), i.e., via stimulation with K562-mbIL21 feeder cells [12]. Understanding the mechanism underlying the vast increase in HLA-DR expression in NK cells may be crucial for the following application of in vitro expanded and activated NK cells for adoptive therapy. In this work, we have characterized the transcriptional patterns of HLA-DR^+^ NK cells and identified possible pathways involved in the induction of HLA-DR expression.

Firstly, we emphasize the main differences between circulating HLA-DR-positive and negative NK cells in two fractions, where the increased expression of HLA-DR was previously observed—less differentiated CD56^bright^ cells and highly differentiated adaptive CD56^dim^CD57^+^ NK cells expressing NKG2C [10,12]. Transcriptome profiling of CD56^bright^ NK cells indicates a highly activated proliferative state of HLA-DR-positive cells, evidenced by the increased expression of several G2/M-phase associated genes, including apoptosis-resistance gene BIRC5, in HLA-DR^+^ samples. At the same time, no genes associated with NK cell activation or the induction of HLA-DR expression were upregulated in the CD56^bright^HLA-DR^+^ subset.

The DE analysis of CD57^+^CD56^dim^ NK cells exhibited several other genes differing in expression between HLA-DR-positive and negative cells, some of which may be important for HLA-DR^+^ NKG2C-expressing adaptive NK cells. In HLA-DR^+^NKG2C^+^CD56^dim^ samples of donor 1, downregulated genes included ZBTB16 encoding the promyelocytic leukemia zinc finger (PLZF) transcription factor, which is shown to be reduced in adaptive NK cells [27]. Another gene decreased in HLA-DR^+^ cells is MYOM2, which is commonly enriched in classical NK cells [28].

DE analysis on both donors showed the downregulation of several inhibitory receptors, including KIRs, as well as checkpoints, markers of exhaustion, and a couple of specific markers in HLA-DR^+^NKG2C^+^CD56^dim^ NK cells. Downregulated SH2D1B gene encodes EAT-2, an adaptor protein of activating receptor SLAMF7. A decrease in SH2D1B expression was shown to cause NK cell cytotoxic activity impairment and the acquisition of exhausted phenotypes [35]. In another study, SH2D1B downregulation was observed in adaptive NK cells [36]. Another gene, FCERG1, which encodes the FcRγ adaptor protein for NK cell activating receptors CD16, NKp30, or NKp46, is also commonly downregulated in adaptive NK cells [37]. Interestingly, CD160, which is important for IFNγ production in NK cells, was downregulated too [38]. HAVCR2, which encodes checkpoint receptor TIM-3, marks the terminal differentiation of NK cells [39]. It has been demonstrated that TIM-3 substantially decreases after NK cell activation [40], followed by reduced cytotoxicity and IFNγ production [40]. KLRB1 (CD161), another inhibitory NK cell receptor, characterizes less differentiated NK cells [41]; thus, its downregulation should be associated with a more mature state, as shown for hCMV-associated adaptive NK cells [42]. TMIGD2—an activating NK cell and T cell receptor interacting with HHLA2 [43]—is mostly expressed on naive T cells, while memory cells lose their expression [44]; therefore, their downregulation may be associated with the memory NK cell phenotype. Genes upregulated in the HLA-DR^+^NKG2C^+^ samples mostly included those associated with MHC class II and its regulation (alpha and beta chains of HLAs, CIITA), as well as several functional genes: CCR5, a chemokine receptor, is usually upregulated during infection and it is important for NK cell recruitment to inflamed tissue [45]; CD34 is a marker of immature progenitor cells [46]; and STYK1 is serine/threonine/tyrosine kinase 1, which shares its homology with fibroblast growth factor receptors and may regulate cell proliferation and survival [47]. Interestingly, the DE analysis of the transcriptomes of NKG2C^+^ vs. NKG2C^−^ NK cells also showed the upregulation of certain MHC class II molecules and CIITA [27]. The overall gene expression pattern of the HLA-DR^+^NKG2C^+^ subset indicates a more activated, adaptive-like state compared to HLA-DR^−^NKG2C^−^CD57^+^CD56^dim^NK cells; however, the downregulation of several inhibitory self-controlling receptors may be associated with the attenuation of functional activity, as in the case of uneducated NK cells.

Before investigating of the exact CIITA isoforms involved in HLA-DR expression in NK cells, we assumed that this might be either the isoform 4 transcribed from the IFNγ-inducible pIV promoter or T-cell-associated isoform 3 from the pIII promoter, due to the similarity of NK cells in terms of origin and some aspects of biology with T cells. Then, we demonstrated the transcription of isoform 3 in HLA-DR^+^ NK cells acquired via pre-activation with IL-2 and K562 cells expressing IL-21. Thus, the results favor the involvement of pIII promoter, similar to activated T cells.

In general, according to RNA sequencing data, HLA-DR^+^ NK cells from a less differentiated subpopulation (CD56^bright^) and a more differentiated subpopulation (CD56^dim^CD57^+^NKG2C^+^) represent cell subsets with significantly different expression patterns and, presumably, functional capacities. CD56^bright^ HLA-DR^+^ NK cells can be characterized as being at or ready for the stage of active proliferation. NK cells CD56^dim^CD57^+^ HLA-DR^+^NKG2C^+^ demonstrate a tendency toward mobility and activation, escaping tolerance and the upregulation of a wider range of components of the antigen-presenting system.

To describe the metabolic activity of HLA-DR-expressing NK cells, we conducted transcriptomic analysis and metabolic in vitro assays. GO analysis of transcriptome revealed the activation of mitochondrial respiratory chain complex IV in HLA-DR^+^NKG2C^+^CD57^+^CD56^dim^ NK cells. In functional tests, the fraction of HLA-DR-positive NK cells demonstrated a higher ATP level and higher mitochondrial mass both ex vivo and after activation when compared to HLA-DR-cells. It is known that a high level of ATP production and an increased volume of mitochondrial mass are characteristic of activated NK cells [24]. This metabolic restructuring allows NK cells to launch a full functional response: the production of IFNγ and the implementation of cytotoxic activity [23,24]. Moreover, adaptive NK cells, as a subpopulation with special functional properties, are also characterized by increased levels of mitochondrial mass and mitochondrial potential, as well as oxidative phosphorylation [30]. Accordingly, the HLA-DR-expressing NK cells studied in our work demonstrate an activated metabolic state and are more “prepared” to realize their functional potential.

Previously, we have shown that the stimulation of NK cells with IL-2 and a combination of IL-2+IL-21 results in increased HLA-DR expression [12]. In this work, we have revealed that rhIL21R inhibited the expansion of HLA-DR^+^ NK cell fraction by abrogating IL-21-IL-21R interaction. Thus, signaling from IL-21R is essential for HLA-DR expression. Interestingly, a reduced HLA-DR^+^ NK cell percentage was also detected after the addition of IL21R inhibitor to samples stimulated only with IL-2. CD4^+^ T cells and NKT cells are shown to produce IL-21 in response to IL-2 stimulation [48], but there are no such data for NK cells. Our results may indirectly indicate that activated NK cells are also able to produce IL-21 to a certain extent. However, this hypothesis needs further investigation.

According to the literature and our previous results, HLA-DR expression in NK cells is often associated with INFγ production [7,12,13]. In addition, transcription from one of the promoters of the CIITA, main MHC class II regulator, is induced precisely under stimulation with IFNγ [16]. In this regard, we hypothesized that HLA-DR expression in NK cells may be induced by IFNγ synthesized by NK cells themselves in response to IL-2 and IL-21 stimulation, apart from direct IL-2- or IL-21-mediated induction. Both of these cytokines are known to enhance the production of IFNγ by NK cells [7,17,31]. Our experiments on IFNγR inhibition confirmed that the signal coming from IFNγ-IFNγR ligation is essential for stimulating an increase in HLA-DR expression on the surface of NK cells. The induction of HLA-DR alpha chain gene expression via IFNγR1-STAT1-dependent activation of the CIITA was described for certain cell lines. However, we were not able to detect the expression of CIITA isoform IV, characteristic of cells responding to IFNγ stimulation, in HLA-DR-positive NK cells, even though HLA-DR expression was clearly dependent on IFNγ–IFNγR interaction, according to our data. Possibly, the induction of CIITA expression in NK cells is mediated through IFNγR independent of the JAK1/3-STAT1 axis and the activation of CIITA promoter IV, for example, through the Ras/Raf/Mek/ERK1/2-mediated pathway. It is known that CIITA is one of the targets of ERK1/2 kinase activity: ERK1/2 phosphorylates CIITA, which leads to its mono-ubiquitination, translocation into the nucleus, and the subsequent induction of MHC II expression [49]. Our results show that the participation of ERK1/2 is essential for the induction of HLA-DR expression on NK cells, supporting the hypothesis concerning independence from STAT1 and are consistent with the published data on the regulation of CIITA expression.

To date, we have found no published confirmation of a direct effect of STAT3 on the activity or expression of CIITA. However, one study suggests that STAT3 is responsible for thyreotropin-induced inhibition of IFNγ-mediated CIITA activation; that is, STAT3 activation inhibits the IFNγ-induced increase in MHC II expression by blocking the STAT1 pathway [50]. In our work, in the presence of IL-21 and low doses of STAT3 inhibitor, there was a tendency toward increased MHC II expression. Possibly, in accordance with the mentioned article, the mild inhibition of STAT3 “released” signal transduction through STAT1, which led to the slight upregulation of HLA-DR expression. However, with an increase in the concentration of STAT3 inhibitor, the expression of HLA-DR significantly decreased, as two of the three possible signal-transducing pathways became interrupted: STAT3 due to direct blockade and STAT1 due to the downregulation of IFNγ synthesis, while the remaining MAPK cascade is only mildly induced by IL-21.

The observed results of the inhibition experiments confirm our assumption that a synergy of signals from exogenous IL-21 and endogenous IFNγ takes place inside NK cells. On the one hand, IL-21 induces IFNγ production and, at least in part, directly influences HLA-DR expression through STAT3 and ERK1/2, which probably induce promotor III of CIITA; on the other hand, IFNγ auto-stimulation augments HLA-DR expression through the same ERK1/2 axis, while STAT1 axis operating via promotor VI of CIITA [20] might be blocked by activated STAT3 [50] and, thus, is not essential for HLA-DR induction in NK cells.

Even though we have not revealed the purpose of HLA-DR expression on NK cells, several research groups have shown the ability of HLA-DR^+^ NK cells to process and present specific antigens on their surface and, as a result, stimulate the activation and proliferation of specific T cells [10,11,51]. In addition to inducing HLA-DR in NK cells, IL-21 regulates immunoglobulin production and, as previously shown, can suppress IgE production [52]. Recently, allergen-specific Th2 cells have been shown to play a significant role in allergic diseases [53]. The question of whether the interaction of HLA-DR-expressing NK cell subsets with CD4^+^ T cells under allergic conditions can further modulate the Th2-associated inflammatory response requires further study.

## 4. Materials and Methods

### 4.1. Cell Lines

A genetically modified clone of the human erythroleukemia cell line K562 expressing membrane-bound IL-21 (K562-mbIL21), additionally expressing tCD19, CD64, CD86, and CD137L, compared with unmodified K562 cells [15], was used for NK cell stimulation.

K562-mbIL21 cells were cultured in RPMI-1640 medium (PanEco, Moscow, Russia) supplemented with 10% FCS (HyClone, Logan, UT, USA), 2 mM L-glutamine (PanEco, Moscow, Russia), and 100-fold Antibiotic Antimycotic Solution (Sigma-Aldrich, St. Louis, MO, USA). Before being used as feeder cells, K562-mbIL21 cells were irradiated (100 Gray), frozen, and stored at −150 °C.

### 4.2. NK Cell Cultures

Blood samples were obtained from 15 healthy adult volunteers. All of the participants gave their informed consent. This study was conducted in accordance with the guidelines of the World Medical Association’s Declaration of Helsinki.

The PBMC fraction was obtained via the gradient centrifugation of blood samples using a standard Ficoll solution (PanEco, Moscow, Russia) with a density of 1.077. NK cells were isolated from PBMC through negative magnetic separation with NK Cell Isolation Kit (Miltenyi Biotec, Bergisch Gladbach, Germany), according to the manufacturer’s protocol. The purity of the freshly isolated NK cells reached 95–99%.

NK cells were cultivated in NK MACS medium (Miltenyi Biotec, Bergisch Gladbach, Germany) and DMEM (PanEco, Moscow, Russia) in a proportion of 1:1 and supplemented with 10% FCS (HyClone, Logan, UT, USA), 2 mM of L-glutamine (PanEco, Moscow, Russia), and Antibiotic Antimycotic Solution (Sigma-Aldrich, St. Louis, MO, USA).

### 4.3. Flow Cytometry

HLA-DR expression in the NK cell subsets was assessed via flow cytometry. Cells were labeled with the following mouse anti-human monoclonal antibodies: CD56-APC clone N901, CD56-PE clone N901, HLA-DR-PE-Cy7 clone Immu-357 (Beckman Coulter, Brea, CA, USA), HLA-DR-FITC clone L243, HLA-DR-PE clone L243, HLA-DR-Brilliant Violet 421 clone L243, CD56-PE-Cy7 clone 5.1H11, CD3-PerCP clone HIT3a, CD56-Brilliant Violet 421 clone 5.1H11 (Sony Biotechnology, San Jose, CA, USA), NKG2C-PE clone REA205, CD57-VioBlue REA769 (Miltenyi Biotec, Bergisch Gladbach, Germany). Viability dye Sytox-Blue (Thermo Fisher Scientific Inc., Waltham, MA, USA) was used to identify the live cells.

The cells were analyzed on a MACSQuant10 flow cytometer (Miltenyi Biotec, Bergisch Gladbach, Germany). The data obtained were processed and presented using FlowJo X 10.0.7r2 (FlowJo LLC, Ashland, OR, USA).

### 4.4. NK Cell Sorting

Ex vivo NK cells were stained with antibodies to CD56, CD3, HLA-DR, CD57, NKG2C. The samples were sorted using the FACSVantage DiVa cell sorter (Beckton Dickinson, Franklin Lakes, NJ, USA) to obtain the desired populations. The purity of sorting exceeded 99%. Samples further used for RNA sequencing were sorted directly into the RLT lysis buffer (QIAGEN, Hilden, Germany).

### 4.5. NK Cell Cultivation with IL-21 Blocker (rhIL-21R) and IFNγR1 Blocker (Anti-IFNγR1 Antibody)

Freshly isolated NK cells at a concentration of 0.5 × 10^6^/mL were cultivated with (a) IL-2, 100 U/mL (Hoffmann-La-Roche, Basel, Switzerland) for the control samples, or (b) IL-2 (100 U/mL) + IL-21, 50 ng/mL (Biolegend, San Diego, CA, USA) for the experiment samples.

The recombinant human soluble fragment of the IL-21 receptor (rhIL-21R) (R&D systems, Minneapolis, MN, USA) was added to the control and all experiment samples at 1, 5, and 15 μg/mL concentrations. IFNγ receptor antibodies (anti-IFNγR1) (R&D Systems, Minneapolis, MN, USA) were added at 0.01, 0.1, and 1 μg/mL concentrations.

NK cells were incubated at 37 °C for 6 days, and the medium with cytokines and blockers was changed on the third day. After 6 days, the cells were labeled with fluorescent antibodies, and marker expression was measured through the use of flow cytometry.

### 4.6. Cultivation of NK Cells with Transcription Factor Inhibitors

The following transcription factor inhibitors were used: fludarabine phosphate (Sigma Aldrich, St. Louis, MO, USA)—STAT1 inhibitor; FR 180204 (Sigma Aldrich, St. Louis, MO, USA)—ERK1/2 inhibitor; and cryptotanshinone (Sigma Aldrich, St. Louis, MO, USA)—STAT3 inhibitor. All substances were diluted to the appropriate concentrations in DMSO (AppliChem, Darmstadt, Germany).

Freshly isolated NK-cells at 0.5 × 10^6^/mL concentrations were cultivated at 37 °C with (a) IL-2, 100 U/mL for the control samples, or (b) IL-2 (100 U/mL) + IL-21 (50 ng/mL) for the experimental samples. On day 3, the medium with cytokines was renewed, and inhibitors were added: fludarabine phosphate, 0.1, 1, and 10 μg/mL; FR 180204, 0.5, 5, and 50 μg/mL; and cryptotanshinone, 0.1, 1, and 10 μg/mL. After 36 h, NK cells were labeled with fluorescent antibodies, and marker expression was measured through the use of flow cytometry.

### 4.7. Evaluation of Mitochondrial Mass Volume

In sorted NK cell subpopulations (HLA-DR^+^ and HLA-DR^−^) ex vivo and 6 days after stimulation with (a) IL-2, 100 U/mL, or (b) IL-2 (100 U/mL) + IL-21 (50 ng/mL), or IL-2 (100 U/mL) + K562-mbIL21 cells (NK:K562 = 5:4) the mitochondrial mass volume were determined via Mitotracker Red FM fluorescent staining (Thermo Fisher Scientific, Waltham, MA, USA), which was performed according to the manufacturer’s protocol. This dye stains active mitochondria in live cells independent of transmembrane potential.

### 4.8. Measurement of ATP Content in NK Cells

ATP production was determined via bioluminescence analysis using a Bioluminescent somatic cell assay kit (Sigma-Aldrich, St. Louis, MO, Germany), according to the manufacturer’s protocol, in sorted NK cell subpopulations (HLA-DR^+^ и HLA-DR^−^) ex vivo and after 6 days stimulation with IL-2 (100 U/mL) + K562-mbIL21 cells (NK:K562 = 5:4). Then, 10^6^ cells per sample were collected, pelleted via centrifugation, added to 400 μL of lysis solution, mixed, and incubated for 4 min at room temperature. The samples were then frozen and stored at −20 °C for further analysis.

The chemiluminescence intensity was measured using a luminometer (Triathler Multilabel tester 425-004, Hidex, Finland). The ATP content in the test sample (in nmol per 10^6^ cells) was calculated in terms of proportion, taking into account the intensity of chemiluminescence and the ATP content in the control sample.

### 4.9. RNA Preparation and Sequencing

The total RNA was extracted from collected samples with TRIzol (Invitrogen, Carlsbad, CA, USA). Treatment with DNase was performed to avoid the influence of genomic DNA. cDNA was obtained with the following amplification using the SMART-Seq^®^ v4 Ultra^®^ Low Input RNA Sequencing Kit according to the manufacturer’s protocol (Takara Bio, San Jose, CA, USA). The resulting cDNA libraries were purified using Ampure XP magnetic beads (Beckman Coulter, Brea, CA, USA) according to the manufacturer’s protocol. Tagmentation and the introduction of indexes was performed using the NexteraXT DNA Library Prep kit (Illumina, San Diego, CA, USA). The resulting libraries were equally mixed and purified using the reagent Ampure XP (Beckman Coulter, Brea, CA, USA). The final library was then subjected to paired-end sequencing on the Illumina NEXTSeq (Illumina, San Diego, CA, USA).

### 4.10. Sequence Analysis

Paired-end RNA-sequencing reads were aligned to the human reference genome (hgGRCh38) using STAR [54]. Unique gene hit counts were calculated using featureCounts [55]. DESeq2 was used to calculate the *p*-value among the samples for each gene [56]. The apeglm method [57] was used to generate *p* values and log2 fold changes; this method provides Bayesian shrinkage estimators for effect sizes in a Negative Binomial (NB) generalized linear model. Genes with absolute log2 fold change (LFC) > 0.58 and *p* adj < 0.1 were considered as DEGs for each comparison. Enrichment analysis was performed using gene ontology (GO) analysis from the clusterProfiler package. The gene list was arranged by LFC (decrescent order). GSEA was run using the gseGO function with default parameters and *p* adj values were obtained via the Benjamini–Hochberg method.

### 4.11. PCR for CIITA Isoform Identification

The cells were lysed using a commercial reagent ExtractRNA (Evrogen, Moscow, Russia). The total RNA was isolated using chloroform, isopropanol, and 75% ethanol. Reverse transcription to obtain cDNA was carried out in a thermal cycler (Applied Biosystems, USA) using a commercial kit containing MMLV revertase (Evrogen, Moscow, Russia) according to the manufacturer’s procedures. The primers used for the reaction were as follows: oligo-dT to total mRNA and specific primer for CIITA gene mRNA (5′ GGTGTCTGTGTCGGGTTCTG 3′) (Evrogen, Moscow, Russia). PCR was performed using polymerase master mix Maxima hot start (Evrogen, Moscow, Russia), with direct and reverse primers (Evrogen, Moscow, Russia) to β-actin (positive control), the α-subunit of HLA-DR and CIITA isoforms that differ in the structure of exon 1 and the untranslated region upstream of it. PCR results were visualized by electrophoresis using an automated gel imaging instrument (BioRad, Hercules, CA, USA).

### 4.12. Statistical Analysis

The data were analyzed using GraphPad Prism 8 (version 8.4.3, GraphPadSoftware, Boston, MA, USA). The statistical significance of the differences in the data with normal distribution was determined via repeated measures one-way ANOVA unless otherwise stated. *p* < 0.05 were considered statistically significant.

## Data Availability

The original data presented in the study are openly available at https://github.com/masha-ust/HLA-DRproject accessed on 18 March 2024.

## Supplementary Materials

The following supporting information can be downloaded at: https://www.mdpi.com/article/10.3390/ijms25094609/s1.
