# Supplementary material for: HLA-DR Expression in Natural Killer Cells Marks Distinct Functional States, Depending on Cell Differentiation Stage
Source: Int J Mol Sci. 2024 Apr 23;25(9):4609. doi: 10.3390/ijms25094609 (PMC11083986; doi:10.3390/ijms25094609)
Supplement: Supplementary file 1 [file ijms-25-04609-s001.zip › ijms-2952846-supplementary.pdf]

Supplementary Material

Supplementary Figures

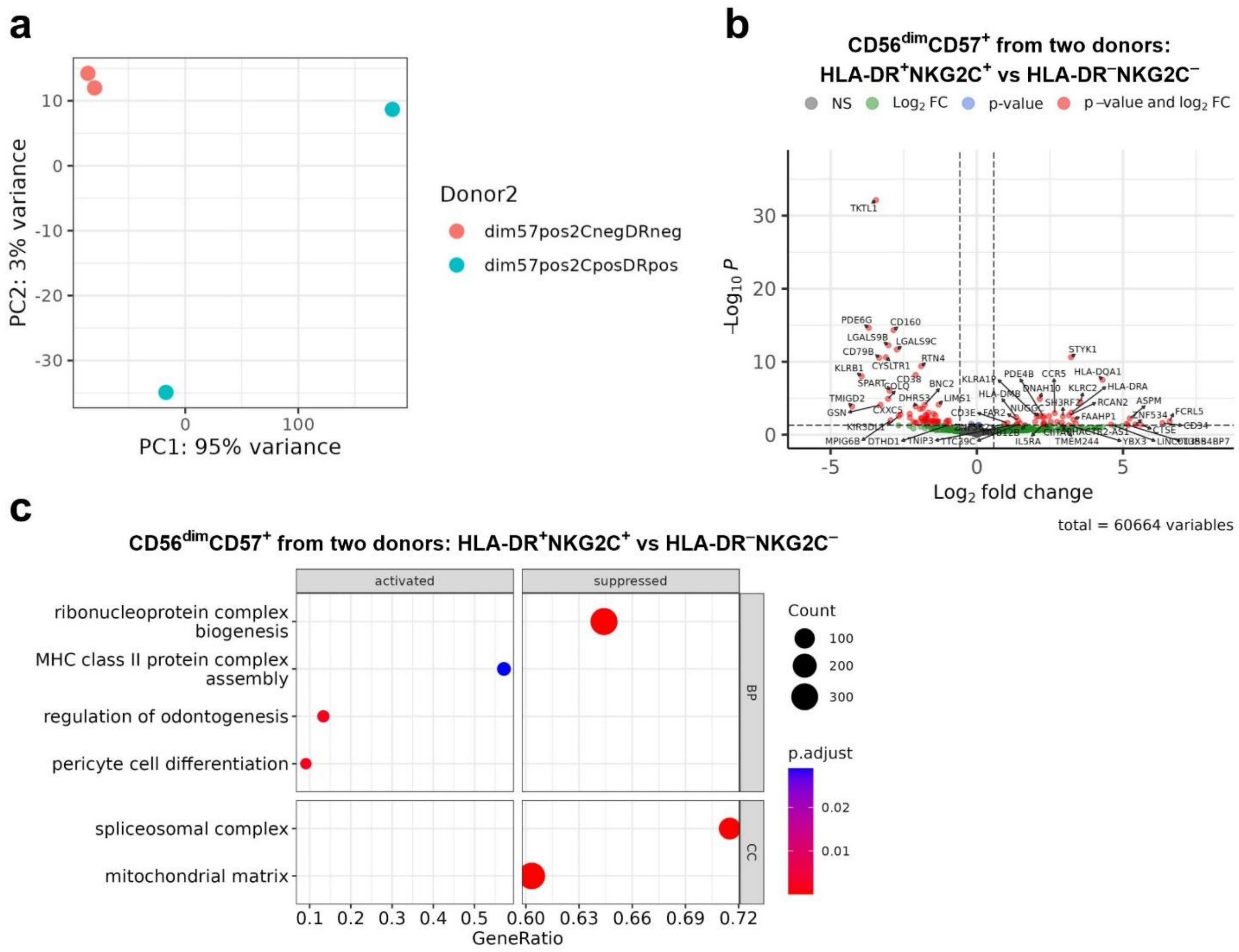

Figure S1. Transcriptome analysis of CD56^{dim}CD57^{+} samples (a) Principal component analysis of normalized count of CD56^{dim}CD57^{+} samples of donor 2; (b) Volcano plot comparing HLA-DR^{+}NKG2C^{+} vs HLA-DR^{-}NKG2C^{-} CD56^{dim}CD57^{+} NK cells from two donors, part of significant up-regulated and down-regulated genes are labeled. (c) Dotplot of GeneOntology gene set enrichment analysis of HLA-DR^{+}NKG2C^{+} vs HLA-DR^{-}NKG2C^{-} CD56^{dim}CD57^{+} NK cells of two donors, where y-axis represents GO molecular pathways (Biological Processes (BP) Cellular Component (CC)), separated by sign and x-axis gene ratio. The greater the size of a circle the greater the number of genes involved in a pathway the circles are colored based on p-adjusted value.
